# Supplementary material for: Resolving the full spectrum of human genome variation using Linked-Reads
Source: Genome Res. 2019 Apr;29(4):635–45. doi: 10.1101/gr.234443.118 (PMC6442396; doi:10.1101/gr.234443.118)
Supplement: Supplemental Material [file supp_gr.234443.118_Supplemental_longranger-2.2.zip › longranger-2.2.tar.bz2!/lr2.2-public/lib/stan/cmdstan/stan/doc/api/html/boost__fpclassify_8hpp_source.html]

Stan: src/stan/agrad/rev/boost\_fpclassify.hpp Source File


|  |  |
| --- | --- |
|  | Stan  2.5.0  probability, sampling & optimization |


- Main Page
- Related Pages
- Namespaces
- Classes
- Files

- File List
- File Members

All Classes Namespaces Files Functions Variables Typedefs Enumerations Enumerator Friends Macros Pages

- src
- stan
- agrad
- rev

boost\_fpclassify.hpp

Go to the documentation of this file.

1 #ifndef STAN\_\_AGRAD\_\_REV\_\_BOOST\_FPCLASSIFY\_HPP

2 #define STAN\_\_AGRAD\_\_REV\_\_BOOST\_FPCLASSIFY\_HPP

3

4 #include <boost/math/special\_functions/fpclassify.hpp>

5 #include <stan/agrad/rev.hpp>

6

7 namespace boost {

8

9  namespace math {

10

22  template <>

23  inline

24  int fpclassify(const stan::agrad::var& v) {

25  return (boost::math::fpclassify)(v.val());

26  }

27

37  template <>

38  inline

39  bool isfinite(const stan::agrad::var& v) {

40  return (boost::math::isfinite)(v.val());

41  }

42

52  template <>

53  inline

54  bool isinf(const stan::agrad::var& v) {

55  return (boost::math::isinf)(v.val());

56  }

57

67  template <>

68  inline

69  bool isnan(const stan::agrad::var& v) {

70  return (boost::math::isnan)(v.val());

71  }

72

82  template <>

83  inline

84  bool isnormal(const stan::agrad::var& v) {

85  return (boost::math::isnormal)(v.val());

86  }

87

88  }

89 }

90 #endif

91

boost::math::isnan

bool isnan(const stan::agrad::var &v)

Checks if the given number is NaN.

**Definition:** boost\_fpclassify.hpp:69

boost::math::isfinite

bool isfinite(const stan::agrad::var &v)

Checks if the given number has finite value.

**Definition:** boost\_fpclassify.hpp:39

stan::agrad::var::val

double val() const

Return the value of this variable.

**Definition:** var.hpp:209

boost::math::fpclassify

int fpclassify(const stan::agrad::var &v)

Categorizes the given stan::agrad::var value.

**Definition:** boost\_fpclassify.hpp:24

boost::math::isinf

bool isinf(const stan::agrad::var &v)

Checks if the given number is infinite.

**Definition:** boost\_fpclassify.hpp:54

rev.hpp

stan::agrad::var

Independent (input) and dependent (output) variables for gradients.

**Definition:** var.hpp:27

boost::math::isnormal

bool isnormal(const stan::agrad::var &v)

Checks if the given number is normal.

**Definition:** boost\_fpclassify.hpp:84


---

[ Stan Home Page ]

*© 2011–2014,
Stan Development Team.*
